# Complications of low compared to standard pneumoperitoneum pressures in laparoscopic surgery for benign gynecologic pathology: a systematic review protocol

**DOI:** 10.1186/s13643-015-0091-6

**Published:** 2015-07-20

**Authors:** Esther B. Kyle, Sarah Maheux-Lacroix, Amélie Boutin, Madeleine Lemyre

**Affiliations:** CHU de Québec—Université Laval Research Center, 2705, boulevard Laurier, Québec, QC G1V 4G2 Canada; Department of Obstetrics-Gynaecology and Reproduction, Université Laval, 2325, rue de l’Université, Québec, QC G1V 0A6 Canada; Department of Social and Preventive Medicine, Université Laval, 2325, rue de l’Université, Québec, QC G1V 0A6 Canada

## Abstract

**Background:**

No definite consensus has been established about the optimal pressure for artificial pneumoperitoneum when performing laparoscopic surgery. It has been postulated that lowering intra-peritoneal pressure levels while performing general laparoscopic surgery would lower surgical complications including post-operative pain, but data remain scarce about significant operative complications. Furthermore, such data is not available for gynecologic laparoscopy. The objective of this systematic review is to compare the frequency and nature of significant operative complications of lower and standard pneumoperiteoneal pressure levels in gynecologic laparoscopic surgery for benign indications.

**Methods/design:**

We will search PubMed, Embase, the Cochrane Library, randomised control trials registries, and reference lists of included articles. Randomised controlled trials comparing different intra-peritoneal pressure levels in women undergoing gynecologic laparoscopic surgery for a non-malignant indication will be eligible. Two reviewers will independently select and review references, extract data, and assess quality from included studies. We will use RevMan5 to calculate risk ratios and their 95 % confidence intervals to compare the frequency of complications according to intra-peritoneal pressure levels. We will perform sensitivity analyses to explore heterogeneity potentially due to various operative characteristics or characteristics of patients.

**Discussion:**

Our results will help identify the optimal intra-peritoneal pressure level in gynecologic laparoscopic surgery and determine if lowering intra-peritoneal pressure levels while trying to achieve lower levels of post-operative pain is an acceptable change of practice according to the frequency and nature of significant complications.

**Systematic review registration:**

PROSPERO: CRD42015020231

**Electronic supplementary material:**

The online version of this article (doi:10.1186/s13643-015-0091-6) contains supplementary material, which is available to authorized users.

## Background

The choice of therapeutic possibilities has increased considerably since the development of minimally invasive procedures. Laparoscopy has been shown to be a great surgical improvement compared to laparotomy [1]. Blood loss reduction, smaller scars, less pain, and shorter hospital stays are a few advantages of laparoscopy over laparotomy [2, 3].

Nevertheless, surgeons are always aiming at improving the quality of care given to their patients. One of the strategies studied over the last few years is to lower the artificial intra-peritoneal pressure level during laparoscopic surgery. Standard pressures equal to or above 12 mm Hg are traditionally used for intra-abdominal laparoscopy [4, 5]. It has been postulated that lowering pressures would reduce surgical complications such as post-operative pain, ventilation issues, and gas embolism [6, 7]. However, this could also result in increased bleeding, poorer visualisation, and exposure of the operative field, which may also have a significant impact on the welfare of the patient. In a recent systematic review, low intra-peritoneal pressures were associated with lower post-operative pain after cholecystectomy [8] but data remain scarce about other significant operative complications. Furthermore, such data is not available for gynecologic laparoscopy, during which the positioning and the operative manipulations are different than during general laparoscopic surgery. The optimal artificial pneumoperitoneum pressure would allow proper visualisation and security level during the surgery while having the fewest intra- and post-operative complications.

To evaluate the association between intra-peritoneal pressure levels during gynecologic laparoscopic surgery and operative complications, we will review randomised controlled trials comparing low (<12 mm Hg) to standard (≥12 mm Hg) intra-peritoneal pressure levels.

## Methods/design

The methodology of this protocol follows the recommendations of the Cochrane Collaboration group [9] and is reported according to the preferred reporting items for systematic reviews and meta-analyses (PRISMA) group [10] (see Additional file 1).

### Eligibility and criteria

All randomised controlled trials comparing at least two different intra-peritoneal pressure levels during gynecologic laparoscopy and reporting data on any complication, length of surgery, or length of hospital stay will be eligible (Additional file 2). No restriction based on language or publication date will be applied. Considering the need for more invasive surgery during the staging of potentially metastatic diseases and the obvious increased morbidity associated with these diseases, trials including more than 20 % of malignant indications for laparoscopy will be excluded. A malignant indication is defined as a potentially metastatic disease.

### Information sources

The search will be conducted throughout Medline (via PubMed), Embase, and the Cochrane Library from their inception up to a maximum of 6 months before the submission of the manuscript for publication. The reference lists of the selected publications will be sought to possibly identify more trials and systematic reviews on the subject. We will also search randomised controlled trials registries (www.isrctn.com, clinicaltrials.gov) in order to identify additional studies.

### Search strategy

An information specialist was consulted to elaborate the search strategy, and all authors reviewed it. The strategy used for Medline is available in Additional file 3. Keywords and index terms related to laparoscopy and artificial peritoneal pressures were used. Validated filters [11, 12] are used to identify randomised controlled trials. We will use EndNote X7.3 to manage the references and eliminate duplicates.

### Study selection

Two authors will independently screen all references according to the eligibility criteria. The first selection step will consist in a review of titles and abstracts. Full text of remaining references will subsequently be revised to assess eligibility. When necessary, a third reviewer will be involved to solve any disagreement. A PRISMA flow diagram will be provided.

### Data collection process

Two authors will independently use a standardised data extraction sheet. A pilot version is available (see Additional file 4). We will test it with two (2) randomly selected included publications and refine it accordingly. Authors will be contacted if relevant data is missing. When necessary, a third reviewer will be involved to resolve any disagreement. In case of double publication of the same study, data will be combined to consider as one study.

### Data items

Data that will be extracted from each trial include characteristics of the trial study design, characteristics of the participants (age, body mass index, surgery indication), characteristics of the intervention (type of surgery, intra-peritoneal pressures, positioning of the patient, emergency, or elective surgery), and outcomes (blood loss, hemorrhage, organ injury, open surgery conversion, anesthetic complications, re-intervention, re-admission, mortality, length of surgery, length of hospital stay, post-operative pain, conversion from low to standard pressure, and any other complication reported by authors). Intra-peritoneal pressures will be classified as low (<12 mm Hg) or standard (≥12 mm Hg).

### Risk of bias assessment

To assess the risk of bias in individual studies, the Cochrane Collaboration’s tool for assessing risk of bias [13] will be used regarding the primary outcome (as shown in the data collection sheet, Additional file 4). Two reviewers will independently apply the tool to each study included in the review, and a third reviewer will be involved if needed to solve any disagreement. We will consider a study at high risk of bias when one criterion or more is of “unclear risk” or “high risk”. Since blinding of personnel is not possible intra-operatively, this aspect will not be considered in the overall risk of bias. A sensitivity analysis will be performed to compare the low risk of bias to those associated with a “high risk” or “unclear risk” of bias.

### Outcomes

As any type of complications reflect an uncertainty regarding the safety of an intervention and assuming variations in the reporting of complications, we considered the frequency of any intra- or post-operative complications as our primary outcome. This outcome variable will be a composite measure of the number of patients with any complications or else with at least one specific complication reported by authors. The secondary outcomes will be specific-patient-oriented complications, i.e., hemorrhage, organ injury, open surgery conversion, anesthetic complications (such as arrhythmia, severe hypotension, severe oxygen desaturation, etc.), mortality, blood loss, post-operative pain, post-operative re-intervention, re-admission after discharge from hospital, and length of surgery, length of hospital stay, quality of exposure, and need for rising intra-peritoneal pressure level.

### Summary measures

Frequencies of complications as a composite issue according to intra-peritoneal pressure levels will be compared through risks ratios and their 95 % confidence intervals (CI). We will also compute risk ratios for each type of complication (including hemorrhage, organ injury, open surgery conversion, anesthetic complications, re-intervention, re-admission, mortality, other reported complications). The continuous variables such as length of surgery, length of hospital stay, and blood loss will be evaluated with mean differences and their 95 % CI. Post-operative pain being possibly measured on various scales, standardised mean differences, and their 95 % CI will be used to evaluate this outcome.

### Synthesis of results

Analyses will be conducted in an intent-to-treat approach. Meta-analyses will be performed using random-effects models within the Cochrane statistical package RevMan5 (Computer program, Version 5.3. Copenhagen: The Nordic Cochrane Centre, The Cochrane Collaboration, 2014). Risk ratios and their 95 % CI will be pooled using Mantel-Haenszel method. Mean differences and standardised mean differences will be pooled with inverse variance method. In case of rare events, as expected for mortality, analysis will be done using Peto odds ratios. Heterogeneity between included trials will be assessed with the *I*^2^ [14].

### Additional analysis

After discussions with field experts, we plan to conduct sensitivity and subgroup analysis determined a priori, to explore sources of statistical heterogeneity. Sensitivity analyses according to studies with low risk of bias will be conducted. It is known and understood that the Trendelenburg position, obesity, and the pneumoperitoneum can affect the anesthetic management during abdominal laparoscopy [15–17]. The Trendelenburg position is usually used during gynecologic surgery so that bowels move toward the head of the patient, thus out of the pelvis, allowing better visualisation and security. For this reason it is possible that all studies found report surgeries performed in that position, but if not, it could influence the effect measure. Also, because of the often vital situation in which they take place, emergency surgeries are more prone to complications and higher pressure levels might be more beneficial than in elective surgery. In addition, as mini-laparoscopy uses smaller trocarts and instruments, it might have an influence on the association between pressure levels and complications. Therefore, we also plan to conduct subgroup analyses, if sufficient data are available, according to Trendelenburg position vs. other position, mini-laparoscopy vs. other, emergency vs. elective surgery, body mass index <30 vs. body mass index ≥30. Furthermore, additional analyses are planned to compare high intra-peritoneal pressure level (≥15 mm Hg) vs. standard (12 to 14 mm Hg) and high intra-peritoneal pressure level (≥15 mm Hg) vs. low (<12 mm Hg), if data are available.

### Risk of bias across studies

We plan to use the GRADE approach to assess the quality of evidence of this review. The GRADEprofiler (Computer program on www.gradepro.org. McMaster University, 2014) will help us judge the strength of our recommendations.

## Discussion

The determination of the optimal intra-peritoneal pressure level is of great interest for surgeons and their patients [18, 19], and the topic is ought to be studied for gynecologic surgeries. If lower pressures are associated with favorable effects in terms of both success of the surgery and lower number of intra- and post-operative complications, the achievement of such pressures must be sought. On the other hand, if the use of lower pressures is shown to be harmful, surgeons ought to use standard levels of pressure. Therefore, our review will help in establishing optimal practices in terms of intra-peritoneal pressure levels in laparoscopy.

We do expect heterogeneity in study samples and surgery techniques, including different pressure levels being tested. However, we will consider many clinical and methodological variations through sensitivity analyses in order to evaluate the robustness of our findings. Sensitivity analysis according to the risk of bias and grading evidence will also allow the evaluation of the quality and strength of knowledge synthesised by this review. Our review will thus gather current evidences and help guide both future practices and development of studies to further investigate the impact of pressure levels on patient-oriented clinical outcomes.

## Additional files

Additional file 1:
**Prisma checklist.** Items that will be described in the report of this systematic review. Additional file 2:
**Eligibility criteria.** A table showing detailed inclusion and exclusion criteria using the PICOS framework. Additional file 3:
**Search strategy—PubMed.** The search strategy used for Medline, given as an example of the search strategies of this systematic review. Additional file 4:
**Data extraction sheet.** The pilot version of the date extraction sheet that will be used for this systematic review.

## Abbreviations

CI: confidence interval
mm Hg: millimeter of mercury
PRISMA: preferred reporting items for systematic reviews and meta-analyses
