# Supplementary material for: Complications of low compared to standard pneumoperitoneum pressures in laparoscopic surgery for benign gynecologic pathology: a systematic review protocol
Source: Syst Rev. 2015 Jul 20;4:96. doi: 10.1186/s13643-015-0091-6 (PMC4506768; doi:10.1186/s13643-015-0091-6)
Supplement: Additional file 2: — Eligibility criteria. A table showing detailed inclusion and exclusion criteria using the PICOS framework. [file 13643_2015_91_MOESM2_ESM.pdf]

Additional file 2: Eligibility criteria

	INCLUSION CRITERIA	EXCLUSION CRITERIA
Population	Women undergoing intra-abdominal laparoscopy of the female reproductive system (uterus, ovaries, fallopian tubes, vagina) of any type.	More than 20% of malignant (potentially metastatic) diseases.<br>Non-gynecologic interventions.
Intervention	Low (< 12 mm Hg) intra-peritoneal pressure levels.	
Comparison	Standard/higher (≥ 12 mm Hg) intra-peritoneal pressure levels.	
Outcomes	Primary outcome: operative complications (such as detailed in the *Outcomes* section).<br>Secondary outcomes: each operative complication, blood loss (quantity), conversion from low to standard pressure, post-operative pain, re-intervention, re-admission, length of surgery and hospital stay.	
Study design	Randomised controlled trials.	
