# Supplementary material for: Complications of low compared to standard pneumoperitoneum pressures in laparoscopic surgery for benign gynecologic pathology: a systematic review protocol
Source: Syst Rev. 2015 Jul 20;4:96. doi: 10.1186/s13643-015-0091-6 (PMC4506768; doi:10.1186/s13643-015-0091-6)
Supplement: Additional file 3: — Search strategy—PubMed. The search strategy used for Medline, given as an example of the search strategies of this systematic review. [file 13643_2015_91_MOESM3_ESM.pdf]

Search strategy – PubMed (2015-05-05)		
**1.**	("Pneumoperitoneum"[Mesh] OR Insufflation pressures[Title/Abstract] OR Insufflation pressure[Title/Abstract] OR Pneumoperitoneum[Title/Abstract])	7065
**2.**	("Endoscopy"[Mesh] OR “Laparoscopy"[Mesh] OR Laparoscopic[Title/Abstract] OR Laparoscopy[Title/Abstract] OR Endoscopic[Title/Abstract] OR Endoscopy[Title/Abstract])	352388
**3.**	(Animals [Mesh] NOT Humans [Mesh])	3996446
**4.**	#1 AND #2 NOT #3	2808
**5.**	Clinical trial [pt] OR Randomized [ti/ab] OR Placebo [ti/ab] OR Clinical trials [mh] OR Randomly [ti/ab] OR Trial [ti]	1041180
**6.**	#4 AND #5	448
